# Research on the clinical factors of cardiac iron deposition in children with beta-thalassemia major

**DOI:** 10.1007/s00431-023-05300-w

**Published:** 2023-11-08

**Authors:** Yuhang Zhou, Yaxuan Cao, Zhenhua Fang, Ken Huang, Mengxin Yang, Guanxiu Pang, Jie Zhao, Yang Liu, Jianming Luo

**Affiliations:** Department of Pediatrics, The First Affiliated Hospital Of Guangxi Medical University, Nanning, China

**Keywords:** Children, β-thalassemia major, Cardiac iron deposition, Clinical factors

## Abstract

Magnetic resonance imaging (MRI) T2* is the gold standard for detecting iron deposition in cardiac tissue, but the technique has limitations and cannot be fully performed in paediatric thalassemia patients. The aim of this study was to analyse clinical data to identify other predictors of cardiac iron deposition. A retrospective analysis was performed on 370 children with β-TM. According to the cardiac MRI results, patients were allocated to a cardiac deposition group and noncardiac deposition group. Multivariate analysis revealed that genotype and corrected QT interval were associated with cardiac iron deposition, indicating that the-β^0^/β^0^ genotype conferred greater susceptibility to cardiac iron deposition. Receiver operating characteristic curve (ROC) analysis was performed, and the area under the curve (AUC) of genotype was 0.651. The AUC for the corrected QT interval was 0.711, at a cut-off value of 418.5 ms. ROC analysis of the combined genotype and corrected QT interval showed an AUC of 0.762 with 81.3% sensitivity and 64.7% specificity. Compared to patients with the β^+^/β^+^ and β^0^β^+^ genotypes, β^0^β^0^ children with β-TM were more likely to have cardiac iron deposition.

*  Conclusion*: The genotype and QTc interval can be used to predict cardiac iron deposition in children with β-TM who are unable to undergo MRI T2 testing.

**Supplementary Information:**

The online version contains supplementary material available at 10.1007/s00431-023-05300-w.

## Introduction

β-TM is a chronic haemolytic anaemia. High power circulation increases cardiac deposition. Endothelial dysfunction, intimal remodelling and peroxidative damage due to cellular hypoxia and iron overload can lead to myocardial ischaemia and tissue fibrosis, resulting in cardiac injury [1, 2]. Even in paediatric β-TM patients, manifestations of cardiac damage, such as LV dilatation and LV diastolic dysfunction can occur [3]. Cardiac iron deposition is a major cause of death in β-thalassemia major (β-TM). Early diagnosis and treatment can reverse and improve patient survival.

MRI T2* is a noninvasive and highly sensitive test that is the gold standard for the early detection of iron deposits in the cardiac tissue [4–9]. Cardiac MRI T2* can detect and quantify the iron deposition in the cardiac. Survival rates of transfusion-dependent TM patients have improved significantly since the use of MRI T2* techniques [11]. However, this technique requires high levels of physicians and technologists, expensive instruments and equipment and examinations, cannot be carried out in some hospitals, and is challenging for preschool children in terms of cooperation; so MRI T2* cannot be fully implemented in paediatric TM patients.

Therefore, it is crucial to find common clinical indicators that can be used to predict iron deposition in the heart.

## Methods

We retrospectively analysed the clinical data collected from patients with β-TM at the Department of Paediatrics, the First Affiliated Hospital of Guangxi Medical University, between January 2017 and October 2022. The following inclusion criteria were employed: (1) age range 3 to 14 years and (2) cardiac MRI T2* and genetic testing for TM. The following exclusion criteria were employed: (1) patients with infection, (2) severe malnutrition, (3) TM in combination with other inherited haemoglobin disorders and (4) TM in combination with diabetes, chromosomal disorders or malignancy.

General data were collected, including sex, age, height, weight, body mass index (BMI), thalassaemia genotypes, hydroxyurea and chelation therapy. First, as our study was a retrospective analysis, blood samples, MRI and genotype data were obtained from hospital laboratories. The blood samples were peripheral blood from the children. MRI method: the pretransplant magnetic resonance imaging (MRI) of all patients was performed using a 3.0 T scanner (Verio, Siemens, Germany), and data were analysed using CMR Tools software (England). Genetic testing for thalassemia was performed in a hospital laboratory. β+ abnormal genes are *IVS -II- 654*, - *28*, *βE, - 29*, - *30*, - *32*, *CAP* and *IVS -I- 5*. β0 abnormal genes are *CD41-42*, *CD17*, *CD71-72*, *Int*, *CD31*, *CD14-15*, *IVS -I- 1*, *CD43*, *CD27-28*, *CD14-15*, *IVS -I- 1*, *CD43* and *CD27-28*. Clinical variables were defined as follows: Hepatomegaly > 2 cm below the right costal margin and splenomegaly > 2 cm below the left costal margin. Cardiac MRI T2* results were recorded. Cardiac T2* is a quantitative measurement of tissue iron content that correlates negatively with tissue iron content and can be used to diagnose the presence of iron deposition in the myocardium. According to the results of cardiac MRI T2* (normal (T2* > 20 ms), mild (14 ms ≤ T2* < 20 ms), moderate (10 ms ≤ T2* < 14 ms) and severe (T2* < 10 ms) [10]), patients were categorized into a cardiac deposition group and a normal group (noncardiac deposition). Laboratory data were collected within 3 months before or after MRI detection. Laboratory indices included routine blood count, liver and kidney function, cardiac enzymes and echocardiography and abdominal ultrasound. Specifically, routine blood indicators included white blood cell (WBC) count, red blood cell count, platelet count, haemoglobin concentration, mean cellular volume(MCV), mean cellular haemoglobin (MCH), mean corpuscular haemoglobin concentration (MCHC) and red blood cell distribution width (RDW); liver function indicators included total bilirubin, direct bilirubin, indirect bilirubin, total bile acids, alanine aminotransferase (ALT) and aspartate aminotransferase (AST); indicators of cardiac enzymes included creatine kinase (CK), creatine kinase isoenzyme MB (CK-MB) and lactate dehydrogenase; ECG indicators included heart rate, PR interval duration, QRS interval duration, QT interval duration and corrected QT interval; cardiac ultrasound indicators include left ventricular end-diastolic diameter (LVEDD), left ventricular end-systolic diameter (LVESD), right ventricular internal diameter (RVID), left ventricular (LV) fractional shortening, LV ejection fraction, stroke volume, cardiac output and LV end-diastolic volume.

Data were analysed using SPSS 26 software. Data with a normal distribution were expressed as the mean ± standard deviation. Two-group comparisons were performed using the independent *t* test. Data that did not have a normal distribution were expressed as the median (four-digit interval) [P50(P25, P75)]. Two-group comparisons were performed using the Mann–Whitney U test. Proportion data are presented as numbers (percentages). Pearson’s Chi-square test was used to perform multiple group comparisons. To minimize the effect of age on some of the cardiac ultrasound parameters, an analysis of covariance (ANCOVA) was performed with age as the covariate and the measured parameters as the dependent variable. Correlations between variables were analysed by Spearman’s test. Significant indices were analysed using multivariate logistic regression analysis to determine risk factors. The optimum threshold for the significant parameter was constructed using receiver operating characteristic (ROC) curves. Two-sided *P* values < 0.05 were considered significant for all analyses.

## Results

Over the period of observation, a total of 470 patients with β-TM who underwent cardiac MRI T2* were enrolled. One hundred patients were excluded because of incomplete data. Of the 370 patients included, 64 (17.30%) had cardiac iron deposition, and 306 (82.7%) had no cardiac iron deposition. Sixty-two of 64 patients with cardiac iron deposition and 266 of 306 patients with noncardiac iron deposition had combined liver iron deposition. All patients were treated with regular transfusion and iron chelation. The number of cases treated with deferoxamine alone or the combination of deferoxamine and deferasirox was small. Therefore, these two cases of chelation therapy were included in the deferasirox combined with deferoxamine group.

The distribution of age and genotypes differed statistically between the two groups (*p* < 0.001). The differences among the three genotypes were mainly as follows: the incidence of cardiac iron deposition was higher with the β0/β0 genotype than with the β0/β+ (*p* < 0.001) and β+ /β+ (*p* = 0.011) genotypes. There was no statistically significant difference between the incidence of cardiac iron deposition in patients with the β0/β+ and β+ /β+ genotypes (*p* = 0.714). There were no significant differences in sex, BMI, chelation therapy, hydroxyurea therapy, hepatomegaly or splenomegaly between the two groups (*p* > 0.05). The clinical characteristics of the patients are shown in Table 1.

**Table 1:** Comparison of the clinical characteristics between the two groups

	Noncardiac iron deposition (*n* = 306)	Cardiac iron deposition (*n* = 64)	*p* value
Sex, male, *n* (%)	184 (60.13)	38 (59.38)	0.911
Age(year), mean ± SD	8.69 ± 2.69	10.51 ± 2.90	< 0.001
BMI(kg/m^2^), mean ± SD	15.67 ± 1.93	15.59 ± 2.16	0.436
Genotype, *n* (%)			
β^0^/β^0^	139 (45.42)	48 (75.00)	< 0.001
β^0^/β^+^	129 (42.16)	13 (20.31)	
β^+^/β^+^	38 (12.42)	3 (4.69)	
Chelation therapy, *n* (%)			
DFP	109 (35.62)	24 (37.50)	0.932
DFX	87 (28.44)	17 (26.56)	
DFP + DFO	55 (17.97)	10 (15.63)	
DFX + DFO	55 (17.97)	13 (20.31)	
Hydroxyurea therapy, *n* (%)	243 (79.41)	56 (87.50)	0.135
Hepatomegaly, *n* (%)	151 (49.35)	40 (62.50)	0.056
Spleen, *n* (%)			
Normal	97 (31.70)	17 (26.56)	0.681
Splenomegaly	143 (46.73)	31 (48.43)	
Splenectomy	66 (21.57)	16 (25.00)	

*BMI* body mass index, *DFP* deferiprone, *DFX* deferasirox, *DFO* deferoxamine

In terms of laboratory indicators, indirect bilirubin, total bile acids, ALT, AST, CK, CK-MB, lactate dehydrogenase, PR interval, QT interval, QTc interval, LVEDD, LVESD, RVID, stroke volume, cardiac output and LV end-diastolic volume between the two groups were statistically significant. (all, *p* < 0.05). There were no significant differences in other indicators.

The cardiac ultrasound indices LVEDD, LVESD, RVID, stroke volume, cardiac output and LV end-diastolic volume were affected by age. Age was approximately normally distributed in the cardiac iron deposition group and normally distributed in the noncardiac iron deposition group. The age, LVEDD, LVESD, RVID, stroke volume, cardiac output and LV end-diastolic volume of the samples in each group had uniform variance, and the correlation coefficients of the six cardiac ultrasound indices with age were the same, which satisfied the conditions of analysis of covariance. The differences in LVEDD, LVESD, RVID, stroke volume, cardiac output and LV end-diastolic volume between the two groups after correction were not statistically significant (all, *p* > 0.05), as shown in Table 2.

**Table 2:** Comparison of laboratory indicators between noncardiac iron deposition andcardiac iron deposition

	Noncardiac iron deposition (*n* = 306)	Cardiac iron deposition ( n = 64)	*p* value
WBC count [*10^9/L, *P*_50_ *(P*_25_, *P*_75_)]	5.98 (4.60, 8.79)	6.95 (4.68, 9.48)	0.123
Red blood cell count (*10^12/L, mean ± SD)	3.46 ± 0.57	3.36 ± 0.51	0.226
Haemoglobin (g/L, mean ± SD)	87.10 ± 9.00	86.98 ± 10.88	0.927
Platelet count [*10^9/L, *P*_50_ (*P*_25_, *P*_75_)]	297.35 (226.50, 412.75)	354.60 (215.53, 686.03)	0.051
MCV [fl, *P*_50_ (*P*_25_, *P*_75_)]	79.23 (76.15, 81.01)	79.91 (77.87, 81.14)	0.303
MCH (pg, mean ± SD)	24.75 ± 1.92	24.76 ± 1.83	0.976
MCHC (g/L, mean ± SD)	306.55 ± 11.97	304.99 ± 13.32	0.353
RDW [*P*_50_ (*P*_25_, *P*_75_)]	0.19 (0.15, 0.22)	0.19 (0.15, 0.24)	0.485
Total bilirubin [umol/L,* P*_50_ (*P*_25_, *P*_75_)]	20.45 (13.88, 28.70)	17.55 (11.53, 23.93)	0.053
Direct bilirubin [umol/L,* P*_50_ (*P*_25_, *P*_75_)]	5.30 (3.80, 7.33)	4.70 (3.53, 7.00)	0.163
Indirect bilirubin [umol/L,* P*_50_ (*P*_25_, *P*_75_)]	15.05 (9.50, 21.10)	12.95 (7.43, 17.18)	0.044
Total bile acids [umol/L,* P*_50_ (*P*_25_, *P*_75_)]	7.98 (4.78, 13.55)	9.85 (6.13, 15.05)	0.032
ALT [U/L,* P*_50_ (*P*_25_, *P*_75_)]	31.00 (25.00, 42.00)	37.00 (28.00, 50.50)	0.019
AST [U/L,* P*_50_ (*P*_25_, *P*_75_)]	23.00 (13.00, 37.00)	29.00 (18.50, 53.50)	0.027
Creatine kinase [U/L,* P*_50_ *(P*_25_, *P*_75_)]	44.00 (33.00, 63.00)	39.00 (26.25, 48.00)b	0.005
CK-MB [U/L,* P*_50_ (*P*_25_, *P*_75_)]	15.30 (11.00, 19.00)	12.75 (10.00, 16.75)	0.003
lactate dehydrogenase [U/L,* P*_50_ (*P*_25_, *P*_75_)]	188.00 (156.50, 233.00)	162.00 (126.25, 196.75)	< 0.001
heart rate [beats/min, *P*_50_ (*P*_25_, *P*_75_)]	88.00 (81.00, 97.00)	85.50 (74.50, 92.00)	0.063
PR interval [ms,* P*_50_ (*P*_25_, *P*_75_)]	130.00 (122.00, 144.00)	138.00 (124.00, 149.50)	0.015
QRS interval (ms, mean ± SD)	77.83 ± 9.29	79.52 ± 8.46	0.182
QT interval (ms, mean ± SD)	357.28 ± 31.15	380.28 ± 29.62	< 0.001
Corrected QT interval (ms, mean ± SD)	427.83 ± 28.61	450.13 ± 26.67	< 0.001
LVEDD (mm, mean ± SD)	40.67 ± 0.20^a^	40.82 ± 0.45^a^	0.762
LVESD (mm, mean ± SD)	25.21 ± 0.16^a^	25.23 ± 0.35^a^	0.954
RVID (mm, mean ± SD)	15.71 ± 0.13^a^	15.79 ± 0.28^a^	0.816
LV fractional shortening (%, mean ± SD)	38.18 ± 4.02	38.09 ± 4.45	0.875
LV ejection fraction (%, mean ± SD)	68.61 ± 5.05	68.48 ± 5.09	0.861
Stroke volume (ml/B, mean ± SD)	50.45 ± 0.65^a^	51.79 ± 1.45^a^	0.404
Cardiac output (L/min, mean ± SD)	4.51 ± 0.07^a^	4.64 ± 0.15^a^	0.436
LV end-diastolic volume (mL, mean ± SD)	74.22 ± 0.87^a^	74.70 ± 1.93^a^	0.824

*WBC* white blood cell count, *MCV* mean cellular volume, *MCH* mean cellular haemoglobin, *MCHC* mean corpuscular haemoglobin concentration, *RDW* red blood cell distribution width, *ALT* alanine aminotransferase, *AST* aspartate aminotransferase, *CK* creatine kinase, *CK-MB* creatine kinase isoenzyme MB,*LV* left ventricular, *LVEDD* left ventricular end-diastolic diameter, *LVESD* left ventricular end-systolic diameter, *RVID* right ventricular internal diameter

^a^Means after removing age confounders

Statistically different indicators and cardiac MRI T2* values were analysed by correlation, indicators that did not have a linear relationship with cardiac MRI T2* values were excluded, and the indicators entered into the multifactorial analysis had a low degree of correlation (correlation coefficient < 0.5) or were not correlated (Supplementary File 1). Final inclusion of age, genotype, indirect bilirubin, AST, CK-MB, lactate dehydrogenase and corrected QT interval row logistic analysis. Multivariate logistic regression analysis showed that genotype and a high corrected QT interval were associated with cardiac iron deposition, as shown in Table 3.

**Table 3:** Results of logistic regression analyses of cardiac iron deposition

Characteristics	Univariable		Multivariable	
	Odds ratio (95% CI)	*p* value	Odds ratio (95% CI)	*p* value
Age	1.272 (1.147, 1.410)	< 0.001	1.103 (0.972, 1.251)	0.128
Genotype	2.726 (1.635, 4.544)	< 0.001	1.953 (1.122, 3.399)	0.018
Indirect bilirubin	0.963 (0.931, 0.997)	0.034	0.942 (0.902, 0.983)	0.006
AST	1.013 (1.002, 1.024)	0.023	1.014 (1.000, 1.028)	0.053
CK-MB	0.919 (0.869, 0.972)	0.003	0.934 (0.871, 1.002)	0.056
Lactate dehydrogenase	0.992 (0.987, 0.997)	0.003	0.996 (0.991, 1.002)	0.212
Corrected QT interval	1.028 (1.017, 1.038)	< 0.001	1.040 (1.026, 1.054)	< 0.001

*AST* aspartate aminotransferase, *CK-MB* creatine kinase isoenzyme MB

**Fig. 1:**
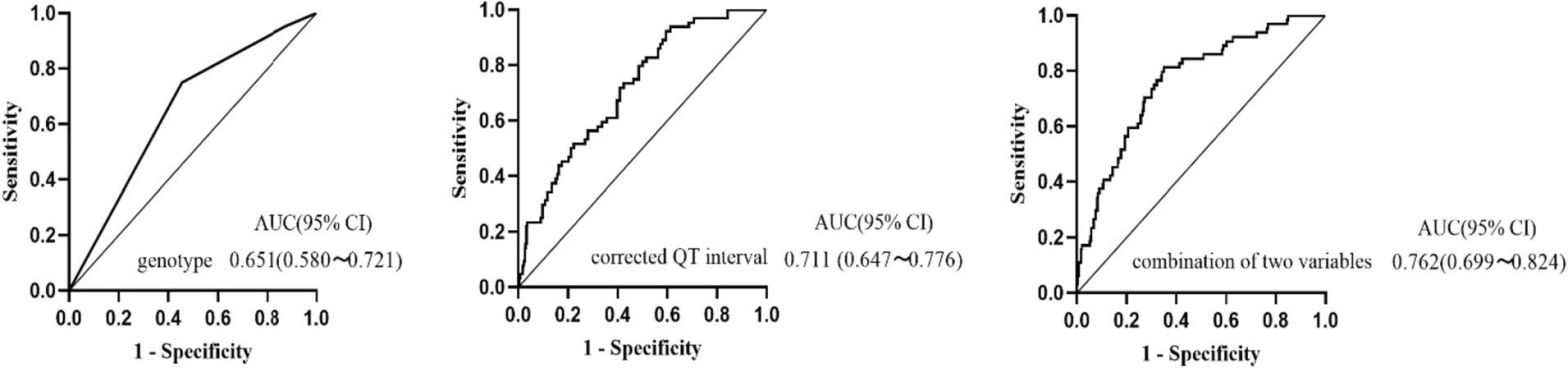
ROC curves of genotype, corrected QTc interval and combination of two variables. ROC, receiver operating characteristic; AUC, area under the curve

Genotype, corrected QT interval and combination of two variables were analysed by ROC curves, and ROC curve results are shown in Fig. 1 and Table 4.

**Table 4:** Area under the ROC curve for genotype, corrected QT interval and combination of two variables

Characteristics	AUC (95% CI)	*p* value	Sensitive (%)	Specific (%)	Cut-off value
Genotype	0.651 (0.580 ~ 0.721)	< 0.001	75.0	54.6	
Corrected QT interval	0.711 (0.647 ~ 0.776)	< 0.001	93.8	37.9	418.5 ms
Combination of two variables	0.762 (0.699 ~ 0.824)	< 0.001	81.3	64.7	

*AUC* area under the curve

## Discussion

β-TM is a classic secondary iron overload disease. Excessive iron deposition in the heart leads to dilated cardiomyopathy and cardiac failure, which are the main causes of death in patients [12–15]. Early identification and treatment of cardiac iron deposition may improve patient prognosis and increase survival. Defects in the β-globin gene, resulting in the absence (β0) or reduction (β+) of β-globin chain synthesis, are characteristic of β-TM. The degree of clinical disease varies among β-TM patients with different genotypes. Patients with major TM require regular blood transfusions for a long time after the onset of the disease, and the older they are and the more frequent the transfusions are, the greater the probability of organ iron deposition occurring. In the present study, we found that the age of patients in the cardiac iron deposition group was higher than that of patients in the noncardiac iron deposition group; in addition, there was a correlation between age and cardiac iron deposition, but increasing age was not a factor that promoted the occurrence of cardiac iron deposition. Differences in genotype can also exhibit varying degrees of cardiac iron deposition. Sagar et al. found that iron toxicity-induced DNA damage was greater in β0 homozygotes than in β0 heterozygotes or β0β+ heterozygotes [16]. Pistoia et al. conducted a cross-sectional study of MRI T2* values and cardiac iron deposition in different genomes of β-TM patients and found that β0 homozygotes and β0β+ heterozygous patients had higher levels of cardiac iron deposition than β+ homozygotes [17]. Another study showed that β0 homozygotes and β0β+ patients were at higher risk of cardiac iron deposition and LV dysfunction [18]. Our study found a higher incidence of cardiac iron deposition with the β0/β0 genotype than with the β0/β+ and β+ /β + genotypes, which is consistent with the above findings. We also found that the β0/β0 genotype promotes the development of iron deposition in the cardiac tissue. However, since β0/β0 patients have a clinical presentation of TM major with an early onset and a high number of transfusions and are prone to iron overload, the effect of the number and volume of transfusions on this conclusion cannot be excluded.

Excessive iron deposition in the liver can lead to liver damage, liver fibrosis, cirrhosis and even hepatocellular carcinoma [19]. Although ALT and AST are mainly used to evaluate liver impairment, ALT and AST play a considerable role in the development of cardiovascular disease [20–22] and are also associated with endothelial dysfunction and coronary artery disease [23]. In the present study, we found that the levels of ALT and AST were higher in the cardiac iron deposition group than in the noncardiac iron deposition group, and there was a negative correlation between ALT and cardiac iron deposition (Supplementary Material 1). However, 62 of the 64 children with cardiac iron deposition in this study also had liver iron deposition, so the effect of liver iron deposition on transaminases cannot be excluded. Therefore, whether elevated transaminases in this study can be used to evaluate iron deposition in the cardiac tissue of children with TM needs further study.

Myocardial enzyme profile indicators such as creatine kinase, creatine kinase isoenzyme and LDH are commonly used in clinical practice to assess myocardial damage. The myocardial enzyme profile tends to increase as the degree of myocardial damage increases. However, few studies have focused on the effects of cardiac iron deposition on myocardial enzyme profiles. In this study, the indices of the myocardial enzyme profile did not increase significantly due to the presence of cardiac iron deposition but were lower than those in the noncardiac iron deposition group, indicating that the presence of cardiac iron deposition in β-TM patients cannot be determined by myocardial enzyme-related indices. Moreover, the cardiac enzyme profile is still affected by multiple factors, such as impaired liver function, skeletal muscle damage and tumours [24–26]. The higher cardiac enzymes in the noncardiac iron deposition group than in the cardiac iron deposition group in this study may be related to the combination of severe liver iron deposition in some cases in the noncardiac iron deposition group.

Common electrophysiological abnormalities in patients with cardiac iron deposition include sinus bradycardia, prolonged PR intervals, prolonged QRS duration, elevated or reduced QRS voltage, elevated or reduced ST segment and premature ventricular and atrial beats [27]. The effect of iron deposition on the electrical activity of the cardiac system can cause arrhythmias in TM patients [28]. Patsourakos et al. found a higher incidence of prolonged PR interval, atrial fibrillation and late potentials in β-TM patients than in healthy adults [29]. In the present data, we found longer PR interval in the cardiac iron deposition group than in the noncardiac iron deposition group, which is the same as the results of the above study. However, there was no linear correlation between PR intervals and cardiac MRI T2* values in the 370 patients in this study. However, prolonged PR intervals in β-TM patients suggest the presence of atrioventricular conduction disturbances. Although first-degree atrial ventricular block by itself does not pose a life-threatening condition, patients with β-TM should have long-term ECG monitoring for early recognition of atrial fibrillation and early treatment [30].

TM patients have a high incidence of prolonged QT interval and sudden cardiogenic death [31–33]. Several studies have shown that prolonged QT interval and QTc interval in β-TM patients are associated with severe cardiac iron deposition [34–36], which is seen to cause prolonged QT interval and QTc interval in β-TM patients. In the present study, the QT interval and QTc interval were significantly longer in the children with cardiac iron deposition than in the noncardiac iron deposition group, and there was a correlation between the QTc interval and cardiac iron deposition, which is consistent with the findings of Aggarwal et al., who found that prolonged QRS duration, QT interval and QTc interval in β-TM patients were associated with decreased cardiac MRI T2* values [37]. This study also found that a prolonged QTc interval could be used to predict cardiac iron deposition with a cut-off value of 418.5 ms. However, because the QTc interval showed a low correlation with cardiac iron deposition in this study, and the AUC area was low, the specificity was only 37.9%. Therefore, whether the QTc interval can be used to predict cardiac iron deposition in β-TM patients remains to be studied in large samples and multicentre studies.

In iron deposition cardiomyopathy, LV diastolic dysfunction usually precedes systolic dysfunction [38, 39]. The early stages of cardiac iron deposition lead to diastolic dysfunction and subsequently restrictive cardiomyopathy. Without early recognition and appropriate iron chelation therapy, the disease can eventually progress to end-stage dilated cardiomyopathy. Cardiac MRI T2* values are the most effective tool to accurately assess myocardial iron overload and help guide iron chelation therapy [9].

Cardiac ultrasound combined with cardiac MRI T2* values are sensitive and specific for the early detection of myocardial function and cardiac iron deposition [40, 41]. However, developing countries and low-income countries lack the appropriate equipment. Therefore, cardiac ultrasound is often used to assess early myocardial dysfunction in paediatric and adult patients with TM [42–44]. In this study, we found that in children with thalassemia major, cardiac ultrasound indices did not change significantly in children with cardiac iron deposition, so cardiac ultrasound may not be useful for the early monitoring of cardiac iron deposition.

In conclusion, by studying the clinical data of children with β-TM, we found differences in age, genotype, liver function, myocardial function, electrocardiogram and cardiac ultrasound between children with cardiac iron deposition and those without cardiac iron deposition. We also found that children with genotype β0/β0 β-TM were more likely to develop cardiac iron deposition than children with genotypes β + /β+ and β0/β+ . It was also found that the PR interval and QTc interval were prolonged in children with cardiac iron deposition compared to those with noncardiac iron deposition. Children with β-TM should have early and long-term ECG monitoring for the early identification and treatment of arrhythmias.

We found that the combined β0/β0 genotype and QTc interval could be used to predict cardiac iron deposition in children with β-TM with a sensitivity of 81.3%. The MRI T2* technique remains the gold standard for detecting iron deposition in the cardiac tissue [45]. We believe that genotype and QTc interval can be used to identify patients at risk for cardiac iron deposition in hospitals without this technology or in children who cannot cooperate. However, it is still necessary to follow up the ECG results periodically and to confirm the diagnosis with MRI when conditions permit.

### Supplementary Information

Below is the link to the electronic supplementary material.

Supplementary file1 (DOCX 25 KB)

## Data Availability

The authors confirm that the data supporting the findings of this study are available within the article and its supplementary materials. Data are available upon reasonable request.
