# Correction to: Research on the clinical factors of cardiac iron deposition in children with beta-thalassemia major

**DOI:** 10.1007/s00431-023-05357-7

**Published:** 2023-11-30

**Authors:** Yuhang Zhou, Yaxuan Cao, Zhenhua Fang, Ken Huang, Mengxin Yang, Guanxiu Pang, Jie Zhao, Yang Liu, Jianming Luo

**Affiliations:** Department of Pediatrics, The First Affiliated Hospital Of Guangxi Medical University, Nanning, China

**Correction to: European Journal of Pediatrics** **https://doi.org/10.1007/s00431-023-05300-w**

In this article the author’s name "Mengxin Yang" was incorrectly written as "Mengxing Yang".

The original article has been corrected.

