# Supplementary material for: Research on the clinical factors of cardiac iron deposition in children with beta-thalassemia major
Source: Eur J Pediatr. 2023 Nov 8;183(2):875–82. doi: 10.1007/s00431-023-05300-w (PMC10912130; doi:10.1007/s00431-023-05300-w)
Supplement: Supplementary file 1 — Supplementary file1 (DOCX 25 KB) [file 431_2023_5300_MOESM1_ESM.docx]

|  | MRI T2^*^ value | Age | Geno-type | indirect bilirubin | total bile acids | ALT | AST | CK | CK-MB | LDH | PR | QT | QTC | LVEDD | LVESD | RVID | stroke volume | cardiac output | LV end-diastolic volume |
| --- | --- | --- | --- | --- | --- | --- | --- | --- | --- | --- | --- | --- | --- | --- | --- | --- | --- | --- | --- |
| MRI T2* value | 1.000 |  |  |  |  |  |  |  |  |  |  |  |  |  |  |  |  |  |  |
| Age | -0.141^**^ | 1.000 |  |  |  |  |  |  |  |  |  |  |  |  |  |  |  |  |  |
| Genotype | -0.179^**^ | 0.102^*^ | 1.000 |  |  |  |  |  |  |  |  |  |  |  |  |  |  |  |  |
| indirect bilirubin | 0.159^**^ | 0.140^**^ | -0.046 | 1.000 |  |  |  |  |  |  |  |  |  |  |  |  |  |  |  |
| total bile acids | -0.018 | 0.020 | 0.023 | -0.017 | 1.000 |  |  |  |  |  |  |  |  |  |  |  |  |  |  |
| ALT | -0.074 | -0.066 | 0.007 | 0.091 | 0.265^**^ | 1.000 |  |  |  |  |  |  |  |  |  |  |  |  |  |
| AST | -0.105^*^ | 0.070 | 0.016 | -0.033 | 0.258^**^ | 0.712^**^ | 1.000 |  |  |  |  |  |  |  |  |  |  |  |  |
| CK | 0.033 | -0.296^**^ | -0.153^**^ | -0.256^**^ | 0.044 | 0.019 | -0.062 | 1.000 |  |  |  |  |  |  |  |  |  |  |  |
| CK-MB | 0.134^**^ | -0.285^**^ | -0.150^**^ | -0.067 | 0.163^**^ | 0.125^*^ | 0.092 | 0.410^**^ | 1.000 |  |  |  |  |  |  |  |  |  |  |
| LDH | 0.149^**^ | -0.302^**^ | -0.144^**^ | 0.117^*^ | 0.085 | 0.146^**^ | 0.052 | 0.412^**^ | 0.323^**^ | 1.000 |  |  |  |  |  |  |  |  |  |
| PR interval | -0.074 | 0.225^**^ | 0.149^**^ | -0.054 | 0.037 | -0.012 | -0.001 | -0.124^*^ | -0.045 | -0.118^*^ | 1.000 |  |  |  |  |  |  |  |  |
| QT interval | -0.152^**^ | 0.453^**^ | 0.167^**^ | 0.073 | -0.001 | 0.067 | 0.101 | -0.236^**^ | -0.107^*^ | -0.282^**^ | 0.170^**^ | 1.000 |  |  |  |  |  |  |  |
| QTc interval | -0.133^*^ | 0.242^**^ | 0.089 | 0.087 | 0.007 | 0.194^**^ | 0.115^*^ | -0.197^**^ | 0.003 | -0.072 | 0.145^**^ | 0.536^**^ | 1.000 |  |  |  |  |  |  |
| LVEDD | -0.092 | 0.656^**^ | 0.082 | 0.142^**^ | 0.051 | 0.050 | 0.097 | -0.245^**^ | -0.222^**^ | -0.178^**^ | 0.216^**^ | 0.355^**^ | 0.224^**^ | 1.000 |  |  |  |  |  |
| LVESD | -0.089 | 0.596^**^ | 0.099 | 0.136^**^ | 0.025 | 0.061 | 0.146^**^ | -0.245^**^ | -0.199^**^ | -0.142^**^ | 0.210^**^ | 0.332^**^ | 0.154^**^ | 0.847^**^ | 1.000 |  |  |  |  |
| RVID | -0.069 | 0.579^**^ | 0.082 | 0.105^*^ | 0.053 | -0.019 | 0.109^*^ | -0.185^**^ | -0.152^**^ | -0.166^**^ | 0.178^**^ | 0.327^**^ | 0.096 | 0.539^**^ | 0.505^**^ | 1.000 |  |  |  |
| stroke volume | -0.079 | 0.610^**^ | 0.099 | 0.137^**^ | 0.047 | 0.046 | 0.062 | -0.233^**^ | -0.248^**^ | -0.175^**^ | 0.214^**^ | 0.359^**^ | 0.249^**^ | 0.924^**^ | 0.655^**^ | 0.496^**^ | 1.000 |  |  |
| cardiac output | -0.055 | 0.443^**^ | 0.031 | 0.198^**^ | 0.095 | 0.117^*^ | 0.133^*^ | -0.204^**^ | -0.179^**^ | -0.034 | 0.172^**^ | 0.184^**^ | 0.287^**^ | 0.781^**^ | 0.524^**^ | 0.374^**^ | 0.830^**^ | 1.000 |  |
| LV end-diastolic volume | -0.086 | 0.661^**^ | 0.096 | 0.152^**^ | 0.043 | 0.048 | 0.098 | -0.251^**^ | -0.233^**^ | -0.170^**^ | 0.235^**^ | 0.373^**^ | 0.233^**^ | 0.980^**^ | 0.835^**^ | 0.542^**^ | 0.945^**^ | 0.781^**^ | 1.000 |

Spearman's correlation analysis was performed on the indices with significant differences as well as on the MRI T2* values, and the results were as follows.

ALT: alanine aminotransferase. AST: aspartate aminotransferase. CK: creatine kinase. CK-MB: creatine kinase isoenzyme MB. LDH: lactate dehydrogenase. LVEDD: left ventricular end-diastolic diameter. LVESD: left ventricular end-systolic diameter. RVID: right ventricular internal diameter. LV: left ventricular

The research of clinical factors of cardiac iron deposition in children with beta-thalassemia major

Yuhang Zhou^1^, Yaxuan Cao^1^, Zhenhua Fang, Ken Huang, Mengxing Yang, Guanxiu Pang, Jie Zhao, Yang Liu, Department of Pediatrics, The First Affiliated Hospital Of Guangxi Medical University，China

Jianming Luo*, Department of Pediatrics, The First Affiliated Hospital Of Guangxi Medical University，China.

[jmluo@aliyun.com](mailto:jmluo@aliyun.com)
